# A Practical Approach to Newborn Screening for Severe Combined Immunodeficiency Using the T Cell Receptor Excision Circle Assay

**DOI:** 10.3389/fimmu.2017.01470

**Published:** 2017-11-08

**Authors:** Monica S. Thakar, Mary K. Hintermeyer, Miranda G. Gries, John M. Routes, James W. Verbsky

**Affiliations:** ^1^Department of Pediatrics, Divisions of Hematology/Oncology, Medical College of Wisconsin, Milwaukee, WI, United States; ^2^Children’s Hospital of Wisconsin, Milwaukee, WI, United States; ^3^Department of Pediatrics, Division of Allergy and Clinical Immunology, Medical College of Wisconsin, Milwaukee, WI, United States; ^4^Department of Pediatrics, Division of Rheumatology, Medical College of Wisconsin, Milwaukee, WI, United States

**Keywords:** severe combined immunodeficiency, T cell receptor excision circles, newborn screening, bone marrow transplantation, antibiotic prophylaxis

## Abstract

Severe combined immunodeficiency (SCID) is a life-threatening condition of newborns and infants caused by defects in genes involved in T cell development. Newborn screening (NBS) for SCID using the T cell receptor excision circle (TREC) assay began in Wisconsin in 2008 and has been adopted or is being implemented by all states in 2017. It has been established that NBS using the TREC assay is extremely sensitive to detect SCID in the newborn period. Some controversies remain regarding how screening positives are handled by individual states, including when to perform confirmatory flow cytometry, what is the necessary diagnostic workup of patients, what infection prophylaxis measures should be taken, and when hematopoietic stem cell transplantation should occur. In addition, the TREC can also assay detect infants with T cell lymphopenia who are not severe enough to be considered SCID; management of these infants is also evolving.

## Newborn Screening (NBS) for Severe Combined Immunodeficiency (SCID): An Effective Screening Program

Numerous studies from individual states, as well as a multi-state summary of NBS results, have been published summarizing the results of SCID screening with the T cell receptor excision circle (TREC) assay (1–10). Several conclusions can be made from these studies. First, the TREC analysis is capable of detecting known genetic causes of SCID, including *IL2RG, RAG1, ADA, IL7R, JAK3, DCLRE1C*, and *CD3D* (4). The percentage of cases of X-linked SCID due to mutations in *IL2RG* (19%) is lower than expected based on prior studies (~50%). In addition to known causes of SCID, a number of infants are detected without genetic causes but with clinically defined SCID (1, 2, 4). Thus, it is important to remember that the lack of a genetic diagnosis does not preclude a diagnosis of SCID. Curative treatment is recommended if the infant has a persistently low T cell count (<300/μl autologous T cells) and poor proliferative response to phytohemagglutinin (PHA) (<10% of normal), which are the diagnostic criteria of SCID. Preliminary data from these studies have been used to generate an incidence of SCID in the US population of approximately 1:60,000 (2, 4, 9). Importantly, there have been no cases of documented SCID that have been missed by NBS with the TREC assay, confirming its high sensitivity for detection. It is important to note that some significant immune deficiencies can be missed by the TREC assay, such as late onset ADA deficiency, bare lymphocyte syndrome due to lack of MHC class II, ZAP70 deficiency, CD40 ligand deficiency, and NF-kappa-B essential modulator deficiency. In these cases, the T cells do develop in the thymus, but the defect occurs after VDJ recombination.

In addition to SCID, the NBS using the TREC assay detects a variety of non-SCID disorders that also result in T cell lymphopenia (TCL) (2, 4, 9). Some of these disorders are known to affect normal T cell development, including 22q11.2 deletion syndrome, ataxia telangiectasia, Nijmegen breakage syndrome, and CHARGE syndrome. Interestingly, a variety of chromosomal disorders not classically thought to affect T cell development have also been detected, including Trisomy 21, Jacobsen syndrome, and other cytogenetic anomalies. Congenital anomalies, including cardiac defects, gastrointestinal malformation, and infants with multiple congenital malformations, can be associated with secondary lymphopenia. Most of these disorders, except for complete DiGeorge syndrome and CHARGE syndrome, typically do not result in T cell counts low enough to be considered SCID.

## Practical Care of Infants with a Positive NBS for SCID

When NBS for SCID is implemented, an algorithm regarding how to care for screening positives is essential to the success of the screening program. Each state that performs screening has its own algorithm, but from our experience and published reports, they all are fairly similar (1, 9, 11–13). In Wisconsin, when an infant fails the NBS test for SCID, the primary care doctor and one of the Clinical Immunologists is contacted, and arrangements are made for confirmatory flow cytometry. The TREC level can be informative as to the urgency of this step, as can the current health of the baby. Our approach differs slightly based on whether an infant presents with other concurrent medical issues (e.g., prematurity, medical disorders) or is an otherwise well full-term baby (Table 1). Also, an absent or very low TREC level (e.g., <20% of the cutoff value for TRECs) in an infant is typically treated with more urgency than an infant with a higher TREC level, as these infants are less likely to have SCID.

**Table 1:** Recommended diagnostic evaluation for suspected severe combined immunodeficiency (SCID)/T cell lymphopenia (TCL).

Test	Frequency	Details/rationale
All patients		
T cell receptor excision circles (TRECs)	At initial visit and every 2 weeks if premature or other illness	To confirm results of initial screening test
		To determine if TRECs will normalize once illness or prematurity resolves

Lymphocyte enumeration	At initial visit (if full term and healthy), monthly thereafter	To confirm initial result of screening test. Minimum flow panel includes numbers of CD4 and CD8 T cells, CD56 NK cells, and CD19 B cells. CD45RA and CD45RO analysis to determine the numbers of naïve T cells.

Chimerism testing	OnceTCL is confirmed	To test for material engraftment, infant’s buccal swabs are compared to whole blood using variable number tandem repeats. If male, XX/XY FISH from blood can be performed

T cell mitogens, (e.g., PHA)	Once TCL is confirmed	To test for function of T cells

IgG, IgA, IgM	Once prior to first dose of IVIG/SCIG. IgA and IgM are repeated over time if on IVIG/SCIG	To test for function of B cells

Genetic sequencing of SCID-causing genes	Once severe TCL is confirmed	To establish a genetic diagnosis of SCID, we utilize clinically available SCID panels that cover many SCID-causing genes

DNA duplication/deletion array	Once TCL is confirmed	To rule out 22q11 deletion syndrome, CHARGE caused by deletions, and other chromosomal anomalies

The evaluation of an infant with a failed TREC assay includes a thorough medical history to determine if there were any prenatal exposures or perinatal illnesses that could have contributed to false-positive screening. In some states, premature infants with positive screens are followed clinically, and the TREC assay repeated every 2 weeks until they reach 37 weeks adjusted gestational age, at which time lymphocyte enumeration *via* flow cytometry is performed (2, 9). Other states may perform flow cytometry at an earlier gestational age, as SCID can be diagnosed at this time (5). Similarly, infants with significant infections or congenital anomalies such as cardiac defects are followed and assessed when clinically stable. Chylous effusions or lymphatic malformations can also lead to T cell loss, and flow cytometry should be delayed until the underlying cause is treated. Since secondary lymphopenia is common in these instances, this limits unnecessary costs of repeated lymphocyte enumerations.

For well-appearing full-term infants, the family history is reviewed specifically for young childhood deaths and significant infectious history in siblings and close family members. A social history with focus on caregivers is elicited. A thorough physical exam is performed including evaluation for dysmorphic features, skin rashes, presence of absence of lymphoid tissue, and growth. Laboratory evaluations include lymphocyte enumeration to confirm the diagnosis. A limited flow cytometry panel is performed to enumerate absolute CD4 and CD8 positive T cells, CD56-positive NK cells, and CD19-positive B cells. Naïve and memory T cells are enumerated by CD45RA and CD45RO analyses, respectively. Since maternal engraftment and hypomorphic mutations in SCID-causing genes can result in a higher T cell count than would be expected with typical SCID, it is important to verify whether the T cell repertoire is skewed toward a memory phenotype. To assess for maternal engraftment, chimerism testing using variable number tandem repeats is sent. This can be done by comparing the DNA obtained from the infant’s buccal swabs to whole blood. Typically, there are insufficient numbers of lymphocytes present to perform a sorted CD3 chimerism test. If the infant is a male, a XX/XY chimerism assay by FISH can be performed. If the lymphocyte numbers are consistent with SCID and there is no cause for secondary lymphopenia, these infants are labeled as “presumed SCID” (Table 1).

All infants with presumed SCID then undergo a diagnostic and confirmatory workup during these initial visits. T cell mitogen studies are performed to evaluate T cell function. Genetic testing is pursued to determine if there is a genetic cause for their TCL. Gene panels are cost-effective and preferred for evaluating the many causes of SCID in order to prevent delays associated with single gene testing. DNA deletion/duplication array is performed to exclude 22q11.2 deletion syndrome, microdeletions of CHD7, or other chromosomal anomalies. Due to blood volume issues in an infant, this testing is performed over several visits. It is important to evaluate for the presence of thymic tissue in cases of T-B + SCID, and the genetic tests listed above may not detect all cases of CHARGE or 22q11. Therefore, other imaging can be performed to look for thymic tissue, including a chest ultrasound or a CT scan of the chest. Some centers consider thymic biopsy to confirm the diagnosis of SCID versus athymia (14).

As the diagnostic workup is taking place, evaluation and prophylaxis of infectious diseases are undertaken (Table 2) (5, 11, 12). Baseline infectious disease testing is imperative in all infants suspected of SCID whether symptomatic or not. The frequency of surveillance increases based on clinical suspicion of new infection (e.g., fever, irritability) or if any of the initial results are positive. Infants who test positive for viral infection are hospitalized and anti-viral therapy implemented. Serological assays, with the exception of HIV-1 infection, are not obtained, as specific antibody production is absent in infants with SCID and variably present in infants with TCL. Infants are screened for antibodies to HIV-1, which would indicate maternal HIV infection, and require immediate treatment. We have found that the burden of blood testing is high during this period, with limitations imposed based on patient size. As mentioned above, we typically prioritize infectious disease testing (based on clinical suspicion) over multiple visits.

**Table 2:** Recommended screening tests at initial newborn screening (NBS) referral and pre-hematopoietic stem cell transplantation (HSCT).

Test	At referral for NBS	Monitoring during pre-HSCT period	During pre-transplant evaluation (<3 weeks prior to HSCT)
**Blood PCR tests**			

CMV, EBV, adenovirus, HHV6	X	Every 4 weeks	X
Hepatitis B, hepatitis C, HSV1/2, HIV-1	X		X (omit if performed <30 days prior to HSCT and negative)
NMDP, WNV			X

**Blood antibody testing**			

HIV-1^a^	X		X
NMDP, HIV-2, HTLV 1/2, HepBsAg, Hep B core, Hep C, CMV, *T. cruzi*, STS			X

**Blood serum or plasma**			

Frozen for potential future diagnostic purposes	X		

**Nasopharyngeal swabs for viral PCR and viral culture (regardless of symptoms)**			

Influenza A and B, parainfluenza 1, 2, and 3, RSV, adenovirus, and HMPV	X		X

**Imaging evaluation**			

Chest X-ray; CT scans of head, neck, chest, abdomen, pelvis; urinalysis	Only if clinically indicated to evaluate for infection; if diagnosis is under question, chest ultrasound is informative to evaluate for presence of thymus	Only if clinically indicated	X

*CMV, cytomegalovirus; EBV, Epstein–Barr virus; HHV6, human herpes virus 6; HSV1/2, herpes simplex virus 1/2; HIV-1/2, human immunodeficiency virus 1/2; WNV, West Nile virus; NMDP, National Marrow Donor Program; HTLV 1/2, human T-lymphotrophic virus 1/2; HepBsAg, hepatitis B surface antigen; HepB core, hepatitis B core antibody; T. cruzi, Trypanosoma cruzi antibody; STS, serological test for syphilis; RSV, respiratory syncytial virus; HMPV, human metapneumovirus*.

*^a^HIV-2 testing is not routinely performed as part of the baseline testing due to its low prevalence in the United States; should there be concern for HIV infection, both HIV-1 and HIV-2 should be tested at baseline*.

Infants identified as possible SCID undergo a prophylaxis regimen designed to prevent bacterial, fungal, and viral infections (Table 3) (11, 12). Subcutaneous or intravenous immunoglobulin is started to aid in preventing the acquisition of infections. Infants with SCID do not receive any live vaccinations, and we withhold other routine immunizations as well since these children are on antibody replacement. We instruct family members and close contacts to be up-to-date on immunizations against influenza and pertussis. Although transmission of varicella, measles, and rotavirus through vaccination is also highly unlikely (15), we do not recommend that close contacts or family members be vaccinated with live attenuated vaccines unless there is a community outbreak, or if there are other special circumstances. Our supportive care and infectious disease prophylaxis guidelines are largely based on national consensus guidelines, and this supportive care is continued through the immunologic diagnostic workup until transplant occurs (16). Additionally, complete blood counts and kidney and liver function are followed while on antimicrobials at least every 2–4 weeks. Lymphocyte counts continue to be followed monthly. This is particularly important for those infants whose genetic diagnosis has not yet been confirmed, in order to monitor for any potential for T cell recovery.

**Table 3:** Recommended infectious disease prophylaxis for newborns with suspected of severe combined immunodeficiency.

Prophylaxis	Drug (dose)	Time of initiation	Alternatives	Comments
**In newborn**				

PCP	TMP-SMX orally (5 mg TMP/kg once a day for 2 consecutive days weekly)	1 month old	Atovaquone orally (30 mg/kg once a day)	Verify that bilirubin is <2X’s upper limit of normal before starting. Monitor ALT, AST, and bilirubin every 2–4 weeks
HSV and VSV	Acyclovir orally (20 mg/kg/dose 3 times a day)	At first visit		Follow BUN and creatinine every 2–4 weeks
Respiratory syncytial virus (RSV)	Palivizumab (15 mg/kg I.M.)	1 month old		Given during peak RSV season, typically November–March in the northern hemisphere
General (bacterial/viral)	IVIG (0.4–0.5 g/kg every month) or SCIG	1 month old		Monitor troughs monthly and maintain Ig > 600 mg/dl; Based on subcutaneous fat and body surface area to volume of medication administered, could consider SCIG in select patients
Fungal	Fluconazole (6 mg/kg once daily)	1 month old	Micafungin or discontinue fungal prophylaxis	Follow AST, ALT, and bilirubin every 2–4 weeks

**In family members or close contacts**				

Influenza	Inactivated influenza vaccine	Seasonally		
Pertussis	Tdap vaccine	Per routine childhood vaccinations		One booster for adolescents (11–12 years age); adults 19–64 years age and adults >65 years age

It is during these initial visits that education is provided regarding hematopoietic stem cell transplantation (HSCT). We also request that families maintain continuity with their primary care provider during this time, who can continue monitoring the infant for normal developmental milestones and nutrition. We advise the primary care provider to schedule office visits as the first appointment of the morning, in order to limit waiting room exposures. If not previously done, human leukocyte antigen (HLA) typing is sent on the patient and immediate family members. If blood volume limits are being met due to diagnostic and infectious disease testing, buccal swabs can be used for HLA typing. The transplant service initiates an unrelated donor search when a suitable donor with an HLA match is not found within the family. At our center frequent meetings between the Clinical Immunology and the Bone Marrow Transplant teams occur to discuss any diagnostic dilemmas and to update each other on the patient’s clinical status. Once a diagnosis of SCID is confirmed by genetic testing, or for patients without a genetic diagnosis but with persistent T cell counts below 300 cell/μl and abnormal mitogenic responses, care is formally transferred to Bone Marrow Transplant Service.

If a newborn has any concern for infection, including fever (a one-time fever of 100.4^°^F/38^°^C, or low-grade fevers that do not resolve), admission to the inpatient unit is mandatory. At our center, patients are admitted to the Bone Marrow Transplant Unit in positive pressure rooms. Mandatory hand washing and limited visitation including no sick contacts is strictly implemented. Workup for fevers is broad to include cultures and PCR of the blood, cerebrospinal fluid, and nasopharyngeal swabs with imaging as clinically warranted (Table 2). If the infant is hospitalized, the Infectious Disease service is consulted, as is a Neonatologist or Pediatric Hospitalist for a general pediatrics evaluation.

## Decision of Transplant Timing

Once the diagnosis of SCID has been confirmed, we proceed to curative HSCT using the best available donor. The timing of transplant depends on many factors such as the type of SCID, ability to clear/treat any underlying infections, and the need for and type of conditioning. In some types of SCID caused by DNA repair defects, such as DCLRE1C (artemis) deficiency, ionizing radiation, and certain conditioning regimens can be dangerous, and treatment should aim to minimize exposure to alkylating agents and ionizing radiation (17). In other cases of SCID, such as IL2RG deficiency, HSCT can occur without conditioning and can be performed within the first several months of life (18, 19). Additionally, if there is a concurrent infection, there may be a decision made to proceed to transplant without prior conditioning in order to reduce the risk of transplant-related mortality. However, if conditioning is used, it is our practice to wait until 3–6 months of age to allow for further growth, development, and organ maturity. In patients where the diagnosis has not been confirmed by genetic testing, the severity of TCL is verified through serial testing and the decision to transplant may be delayed until at least 6–9 months of age, if not later, based on evaluation of T cell recovery. In our experience, if an infant remains on prophylactic measures with excellent compliance and close follow-up, short delays in referring to transplant are well tolerated.

## Family Guidance for Infants Detected with SCID/TCL

While the NBS has allowed prompt identification of newborns with SCID/TCL, families confronting this disease are typically overwhelmed with emotion, confusion, and uncertainty when first informed of the result. It is challenging for most families, who see a well-child who is thriving, to consider the underlying concerns of an immune deficiency. Anticipatory guidance and education on preventing infections is essential, although there are controversies regarding this. Some recommendations are routine and should be implemented, including strict hand hygiene, limited exposure to visitors, and no exposure to anyone who is ill. We typically keep these infants at home until transplant as long as the social situation is stable. Other issues are more controversial, such as recommendations regarding breastfeeding and contact precautions, particularly in regards to the chance of acquiring cytomegalovirus (CMV). Our current policy is to immediately halt breastfeeding in any child with absent TRECs until the immune status to CMV of the mother is obtained. CMV can be transferred through saliva or breast milk, so at our center, we test the parents for prior CMV exposure by serology. If the mother is CMV seropositive (IgG or IgM), breastfeeding is discouraged, and formula feeding using boiled water is recommended. Furthermore, if either parent is CMV seropositive, he or she is educated regarding the transmission of this virus through saliva, although we do not insist that families use masks or other contact precautions at home. This is a difficult issue, as we strive to balance the need of families to bond with their newborns, while concurrently preventing any infection exposures that may affect the success of the HSCT.

One of the first jobs of the medical team is to focus on a family’s understanding of the diagnosis and their ability to conform to protective measures and prophylactic antimicrobials, and this can affect disposition of management of the infant (i.e., inpatient versus outpatient). If there are any concerns from a social standpoint in regards to poor care or lack of understanding of prophylactic measures, the patient is admitted for continued management and education. If there are no infectious or social concerns, as mentioned earlier, infants suspected of SCID are cared for at home. We employ several guidelines in order to reduce the risk infection (Table 4). We also require that the infant live within 1-h drive of our hospital, or another appropriate hospital well-versed in taking care of sick children, from where transfer to our hospital would occur should there be a fever or sign of infection. We also send the family home with a handout explaining the diagnosis, current medications, and our contact information should the infant be seen at an outside hospital. A family is educated at the first in-person visit that a high degree of suspicion for infections is critical, because infections can be clinically asymptomatic. With the above precautions, we have had no infants acquire serious or opportunistic infections while awaiting transplant or while immunologic investigations were ongoing.

**Table 4:** Recommended supportive care anticipatory guidelines for infants with suspected severe combined immunodeficiency.

Guideline	Reason
Avoid public places, daycare	Prevent transmission of community-acquired diseases
Limited contact with young children	
Strict hand washing	

No breastfeeding^a^	Prevent transmission of cytomegalovirus (CMV)

Boil ingestible water, including bottled water	Prevent cryptosporidium infection

Avoid all live and live attenuated vaccines (MMR, Varicella, Rotavirus, Flu-Mist)	Prevent infection with vaccine-related viruses

Blood products—leukodepletion and irradiation essential; CMV negative when available	Prevent transmission of CMV and graft versus host disease

*^a^Breastfeeding could be reinitiated if maternal CMV demonstrates no prior exposure (IgG and IgM are both negative, and CMV PCR is negative). If CMV IgG is positive, breastfeeding is strongly discouraged*.

## Idiopathic TCL—A Unique Management Problem for NBS

A number of infants who failed the NBS for SCID are diagnosed with idiopathic T cell lymphopenia (ITL) (1, 9, 20). These patients have low T cell numbers, but not low enough to be considered SCID (>300 autologous T cells/mm^3^), and lack a genetic diagnosis that results in SCID or a known cause of TCL. Evaluation and treatment of these patients is evolving as more is known about this condition. First, it is important to investigate for maternal factors that can influence T cell development. Mothers on immunosuppressants, such as azathioprine, can give birth to infants with lymphopenia that may resolve over time (6, 21). Since hypomorphic mutations in SCID-causing genes can result in severe TCL (also known as leaky SCID), genetic sequencing typically with a “SCID panel” that includes all genes known to cause SCID should be performed (22, 23). With new sequencing technologies, it is possible to screen hundreds of known immune defects that could potentially affect TREC levels, and these studies have led to the description of a variety of genetic defects leading to idiopathic TCL, including *ATM* and *ITK* mutations (6, 24, 25).

T cell counts are followed to determine if the TCL is improving. A controversial issue is what constitutes a “protective” T cell count, or a T cell count that is sufficient to prevent infections. However, the absolute number of T cells is not the only factor, as T cell function also needs to be considered. We perform T cell mitogen studies to determine if the T cells are capable of expanding and obtaining effector function. The acquisition of normal antibody levels in infants with ITL can be informative as a surrogate of T cell function, and we follow IgA, IgM, and IgG. IgG levels will not be accurate once replacement antibody is started, and can be complicated by maternal IgG during the first 4–6 months of life. Protective serum vaccination titers can eventually be helpful, but in the early stages of ITL evaluation, we do not recommend routine immunizations. There are several possible outcomes of patients with ITL. First, the T cell counts may normalize over time, but in our and others experience, the timing of this can vary greatly and may take up to 9 months (9, 20). In other cases, the T cell counts may be persistently low and decrease over time, which would necessitate consideration of proceeding to HSCT (9, 20).

## Summary

Newborn screening for SCID/TCL using the TREC assay has been proven to be extremely effective to detect SCID and other severe forms of TCL, with a sensitivity approaching 100% for SCID. Initial evaluation of infants with a positive TREC assay should include flow cytometry to assess the numbers of naïve T cells, as well as NK cells and B cells, and T cell response to mitogens to evaluate function. Genetic testing is important, and any child with two known variants that cause SCID, undergoes HSCT. Infants with T cell counts not consistent with SCID are followed clinically to see if the TCL will resolve over time. Infectious prophylaxis with antimicrobial medications and immune globulin is important as these children are evaluated. Family counseling is essential, not only to prevent the acquisition of new infections but also to educate the family on the diagnosis and the complexities of HSCT. With these issues in mind, outcomes of children detected with abnormal TREC assays are excellent, as morbidity and mortality has been greatly reduced.

## Author Contributions

MT, JR, JV, and nurse practitioners MH and MG were all involved in the writing and editing of the manuscript.

## Conflict of Interest Statement

The authors declare that the research was conducted in the absence of any commercial or financial relationships that could be construed as a potential conflict of interest.
